# Ocean acidification decreases the light‐use efficiency in an Antarctic diatom under dynamic but not constant light

**DOI:** 10.1111/nph.13334

**Published:** 2015-02-24

**Authors:** Clara J. M. Hoppe, Lena‐Maria Holtz, Scarlett Trimborn, Björn Rost

**Affiliations:** ^1^ Alfred Wegener Institute – Helmholtz Centre for Polar and Marine ResearchAm Handelshafen 12Bremerhaven27570Germany; ^2^ University BremenLeobener Straße NW2‐ABremen28359Germany

**Keywords:** *Chaetoceros debilis*, CO_2_, multiple stressors, photophysiology, phytoplankton, primary production, Southern Ocean

## Abstract

- There is increasing evidence that different light intensities strongly modulate the effects of ocean acidification (OA) on marine phytoplankton. The aim of the present study was to investigate interactive effects of OA and dynamic light, mimicking natural mixing regimes.
- The Antarctic diatom *Chaetoceros debilis* was grown under two pCO_2_ (390 and 1000 μatm) and light conditions (constant and dynamic), the latter yielding the same integrated irradiance over the day. To characterize interactive effects between treatments, growth, elemental composition, primary production and photophysiology were investigated.
- Dynamic light reduced growth and strongly altered the effects of OA on primary production, being unaffected by elevated pCO_2_ under constant light, yet significantly reduced under dynamic light. Interactive effects between OA and light were also observed for Chl production and particulate organic carbon quotas.
- Response patterns can be explained by changes in the cellular energetic balance. While the energy transfer efficiency from photochemistry to biomass production (Φ_e,C_) was not affected by OA under constant light, it was drastically reduced under dynamic light. Contrasting responses under different light conditions need to be considered when making predictions regarding a more stratified and acidified future ocean.

## Introduction

The Southern Ocean (SO) plays a pivotal role in the global carbon cycle (Marinov *et al*., 2006), strongly influencing atmospheric CO_2_ concentrations on glacial–interglacial timescales (Moore *et al*., 2000; Sigman *et al*., 2010). Today, the SO takes up 15–40% of the anthropogenic CO_2_ (Khatiwala *et al*., 2009), a large proportion of which is mediated by phytoplankton, in particular diatoms (Nelson *et al*., 1995; Takahashi *et al*., 2002). The potential for carbon sequestration via the biological pump (Volk & Hoffert, 1985) is, however, restricted through iron and light limitation (Martin, 1990; Moore *et al*., 2007; Feng *et al*., 2010). Regarding the latter, deep vertical mixing induced by strong winds leads to pronounced changes in the light regime as well as low integrated irradiances that phytoplankton cells encounter in the upper mixed layer (MacIntyre *et al*., 2000).

Diatoms tend to dominate under well‐mixed, nutrient‐rich environments where light is the main factor controlling growth rates (Sarthou *et al*., 2005). Even though diatom species were found to differ in their photophysiological characteristics, this group can generally be characterized by high photochemical efficiencies, low susceptibilities towards photoinhibition, and high plasticity in photoacclimation (Wagner *et al*., 2006; Lavaud *et al*., 2007; Kropuenske *et al*., 2009; Su *et al*., 2012; Li & Campbell, 2013). Overall, diatoms seem to be less compromised by fluctuating irradiances than other phytoplankton groups (van Leeuwe *et al*., 2005; Wagner *et al*., 2006; Lavaud *et al*., 2007; Jin *et al*., 2013). These physiological features can, to a large degree, explain the dominance of diatoms in natural phytoplankton assemblages exposed to deep‐mixing regimes like the SO (Sarthou *et al*., 2005). Studies investigating the effects of dynamic light on diatoms often showed that while C : N ratios stayed constant, photosynthetic efficiencies increased and growth rates decreased compared with constant light regimes (e.g. van Leeuwe *et al*., 2005; Wagner *et al*., 2006; Kropuenske *et al*., 2009; Mills *et al*., 2010; Shatwell *et al*., 2012). This indicates increased costs imposed by continuous photoacclimation and/or time spent under nonoptimal configuration of the core physiological apparatus. Despite these general trends, large differences in the magnitude of responses were observed between studies. These could be caused by differences in environmental conditions (e.g. temperatures, nutrient concentrations, seawater carbonate chemistry), which may modulate phytoplankton cells’ ability to cope with fluctuating light fields (Jin *et al*., 2013).

Owing to the high solubility of CO_2_ under low water temperatures (Sarmiento *et al*., 2004), the effects of increased CO_2_ concentrations and decreased pH on SO phytoplankton have gained increasing attention in recent years (Tortell *et al*., 2008; Feng *et al*., 2010; Boelen *et al*., 2011; Hoogstraten *et al*., 2012a,b; Hoppe *et al*., 2013; Trimborn *et al*., 2013). The observed sensitivity of phytoplankton to these changes, commonly referred to as Ocean Acidification (OA), can be partially attributed to beneficial effects of an increased supply of CO_2_. The carbon‐fixing enzyme RubisCO has a poor affinity for CO_2_, with half‐saturation constants (*K*_M_) being higher than the current concentrations of aquatic CO_2_ (Badger *et al*., 1998). To overcome substrate limitation arising from this, phytoplankton employ so‐called carbon concentrating mechanisms (CCMs), which increase the CO_2_ concentration at the reactive site of RubisCO (Reinfelder, 2011). CCMs of diatoms include active CO_2_ and HCO_3_^−^ uptake, C_4_‐like pathways in some species, as well as the expression of carbonic anhydrase, which accelerates the inter‐conversion of CO_2_ and HCO_3_^−^ (Morel *et al*., 1994; Reinfelder *et al*., 2000; Burkhardt *et al*., 2001). Even though CCMs of diatoms were found to be highly efficient in preventing carbon limitation under most conditions (e.g. Badger *et al*., 1998; Hopkinson *et al*., 2011; Trimborn *et al*., 2013), they are also commonly down‐regulated under higher external CO_2_ availability, lowering the overall metabolic costs of carbon acquisition under OA (Burkhardt *et al*., 2001; Rost *et al*., 2003; Trimborn *et al*., 2008).

Results regarding the CO_2_ sensitivity in primary production of diatom‐dominated phytoplankton assemblages as well as isolated strains of the SO vary greatly between studies, indicating little to high potential for ‘CO_2_ fertilization’ (e.g. Tortell *et al*., 2008; Feng *et al*., 2010; Boelen *et al*., 2011; Hoppe *et al*., 2013; Trimborn *et al*., 2014). Such differences in OA responses can be explained by intra‐ and interspecific variability (Langer *et al*., 2009; Trimborn *et al*., 2013), but also by deviating experimental conditions. Besides the impact of temperature (Tatters *et al*., 2013) and nutrient availability (Hoppe *et al*., 2013), the effect of light intensities on OA responses has been shown to be particularly important (Kranz *et al*., 2010; Ihnken *et al*., 2011; Gao *et al*., 2012a). In the coccolithophore *Emiliania huxleyi*, for example, the CO_2_ sensitivity of carbon fixation and calcification was greatly enhanced under low vs high light (Rokitta & Rost, 2012). Several studies on diatoms have shown, furthermore, an increased susceptibility towards photoinhibition under elevated pCO_2_ concentrations (Wu *et al*., 2010; McCarthy *et al*., 2012; Li & Campbell, 2013). Even though all of these studies increased our knowledge on the interactive effects between OA and light intensities, the transferability to processes in the ocean, where light intensities are highly dynamic, is questionable.

Regarding the potential interaction of OA and light regimes, there are only limited data in existence. Boelen *et al*. (2011) did not observe significant effects of pCO_2_ concentrations up to 750 μatm under either constant or dynamic light for the Antarctic diatom *Chaetoceros brevis*. In the coccolithophore *Gephyrocapsa oceanica*, however, the combination of a pCO_2_ of 1000 μatm and short‐term (2 h) exposure to dynamic light led to lowered carbon fixation compared with ambient pCO_2_ and constant light (Jin *et al*., 2013). In view of these conflicting results, a mechanistic understanding of the complex interactions between OA and dynamic light is required. As changes in light harvesting need to be balanced by the sum of all downstream processes, it is particularly important to focus on the interplay between the involved processes and their respective timescales. For example, comparing the short‐term evolution of O_2_ and production of energy carriers and reductive equivalents (ATP and NADPH) with the biomass build‐up or growth on longer timescales clearly shows that both ‘ends of photosynthesis’ do not always match (Behrenfeld *et al*., 2008). Changes in environmental conditions, such as light regime or carbonate chemistry, will inevitably impact the balance of cellular processes, affecting the energy transfer efficiency of photosynthetic light harvesting to carbon fixation (Wagner *et al*., 2006; Rokitta & Rost, 2012).

In view of such considerations and earlier findings on the isolated effects of OA and dynamic light, the goal of the present study was to investigate how the energy transfer efficiency from photochemistry to biomass build‐up and growth is affected by the interaction between OA and dynamic light. To do so, we acclimated the bloom‐forming SO diatom species *C. debilis* to two pCO_2_ concentrations (390 and 1000 μatm) as well as two light regimes (constant and dynamic light), the latter yielding the same integrated irradiance over the day (90 μmol photons m^−2^ s^−1^). This matrix approach was applied in order to test the hypothesis that dynamic light diminishes the beneficial effect of elevated pCO_2_ often observed under constant light, and to understand the physiological mechanisms underlying the general acclimation responses.

## Materials and Methods

### Culture conditions

Monoclonal cultures of the diatom *Chaetoceros debilis* Cleve 1894 (isolated in 2004 by P. Assmy during R/V Polarstern cruise ANT‐XXI/3, European iron fertilization experiment (EIFEX), In‐Patch, 49°36′S, 02°05′E; re‐isolated by C. Hoppe in 2011) were grown in 1 l glass bottles in semicontinuous dilute‐batch cultures (2000–65 000 cells ml^−1^; diluted every 4–5 d) at 3 ± 0.4°C in a 16 : 8 h, light : dark cycle. Media consisted of 0.2 μm sterile‐filtered Antarctic seawater with a salinity of 34 enriched with macronutrients, yielding 180 μmol l^−1^ nitrate, 12 μmol l^−1^ phosphate and 108 μmol l^−1^ silicate. Trace metals and vitamins were added according to F/2 medium (Guillard & Ryther, 1962).

**Figure 1:**
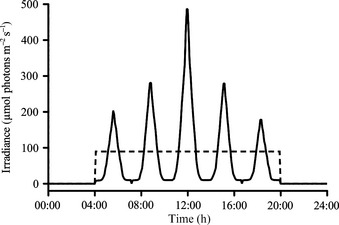
Applied irradiances (μmol photons m^−2^ s^−1^) over the day (16 : 8 h, light : dark cycle) in the constant (dashed line) and dynamic (solid line) light regimes.

For the constant light treatments (Fig. 1), an irradiance of 90 ± 10 μmol photons m^−2^ s^−1^ was applied. Also for the dynamic light treatments (Fig. 1), an average daily irradiance of 90 ± 10 μmol photons m^−2^ s^−1^ was applied. The dynamic light field was calculated assuming a spring situation with a mixed layer depth of 80 m, a mixing speed of 0.014 m s^−1^ (Denman & Gargett, 1983), five mixing cycles d^–1^ and an attenuation coefficient of 0.04 m^−1^, leading to a maximum irradiance of 490 μmol photons m^−2^ s^−1^. The dynamic light modulation (Fig. 1) was controlled via the Control2000 programme of a Rumed incubator (1301; Rubarth Apparate, Laatzen, Germany). In both light treatments, irradiance was provided by identical daylight lamps (Philips Master TL‐D 18W; emission peaks at wavelengths of 440, 560 and 635 nm), thus exposing the phytoplankton to the same spectral composition in all treatments. Light intensities were adjusted by neutral density screens and monitored using an LI‐1400 data logger (Li‐Cor, Lincoln, NE, USA) equipped with a 4π sensor (Walz, Effeltrich, Germany).

Different pCO_2_ conditions were achieved by continuous and gentle aeration of the incubation bottles with air of different CO_2_ partial pressures (390 and 1000 μatm; gas flow rates *c*. 90 ± 10 ml min^−1^) delivered through sterile 0.2 μm air filters (Midisart 2000; Sartorius Stedim, G__tingen, Germany). Gas mixtures were generated using a gas flow controller (CGM 2000; MCZ Umwelttechnik, Bad Nauheim, Germany), in which CO_2_‐free air (< 1 ppmv CO_2_; Dominick Hunter, Kaarst, Germany) was mixed with pure CO_2_ (Air Liquide Deutschland, D__seldorf, Germany). The CO_2_ concentration in the mixed gas was regularly monitored with a nondispersive infrared analyzer system (LI6252; Li‐Cor Biosciences) calibrated with CO_2_‐free air and purchased gas mixtures of 150 ± 10 and 1000 ± 20 ppmv CO_2_ (Air Liquide Deutschland). Cultures were acclimated to treatment conditions for at least 10 generations before sampling, never exceeding 65 000 cells ml^−1^ during this time period.

Continuous aeration with air at the desired CO_2_ partial pressure (390 and 1000 μatm) as well as regular dilution of cultures with pre‐aerated seawater medium led to stable carbonate chemistry over the course of the experiment (Table 1) and to significant differences between pCO_2_ (ANOVA, *P *<* *0.001 for dissolved inorganic carbon (DIC), pH and pCO_2_), but not between light treatments (ANOVA, *P *>* *0.05). In the ambient treatments, pCO_2_ concentrations were 382 ± 18 μatm for constant light and 400 ± 17 μatm for dynamic light. In the OA treatments, pCO_2_ concentrations were 987 ± 28 μatm for constant light and 1026 ± 31 μatm for dynamic light. Over the duration of the experiment (> 5 wk), the drifts in DIC and TA compared with abiotic controls were < 2% and < 4%, respectively.

**Table 1:** Seawater carbonate chemistry was sampled regularly over the course of the experiments (*n *=* *14; mean ± 1 SD)

Treatment	DIC (μmol kg^−1^)	TA (μmol kg^−1^)	pH_total_	pCO_2_ (μatm)
Constant light				
390 μatm CO_2_	2092 ± 15	2250 ± 27	8.05 ± 0.02	382 ± 18
1000 μatm CO_2_	2202 ± 29	2258 ± 18	7.66 ± 0.03	987 ± 28
Dynamic light				
390 μatm CO_2_	2101 ± 27	2263 ± 33	8.03 ± 0.02	400 ± 17
1000 μatm CO_2_	2203 ± 25	2252 ± 27	7.65 ± 0.02	1026 ± 31

DIC, dissolved inorganic carbon. CO_2_ partial pressure (pCO_2_) was calculated from total alkalinity (TA) and pH_total_ at 3°C and a salinity of 34 using CO_2_SYS (Pierrot *et al*., 2006), and concentrations of 12 and 108 μmol kg^−1^ for phosphate and silicate, respectively.

### Carbonate chemistry

Samples for total alkalinity (TA; *n *=* *14) were 0.7 μm filtered (glass fibre filters, GF/F; Whatman, Maidstone, UK) and stored in borosilicate bottles at 3°C. TA was estimated from duplicate potentiometric titration (Brewer *et al*., 1986) using a TitroLine alpha plus (Schott Instruments, Mainz, Germany) and corrected for systematic errors based on measurements of certified reference materials (CRMs provided by Prof. A. Dickson, Scripps, USA; batch no. 111; reproducibility ± 5 μmol kg^−1^). DIC (*n *=* *14) samples were filtered through 0.2 μm cellulose‐acetate filters (Sartorius stedim) and stored in gas‐tight borosilicate bottles at 3°C. DIC was measured colorimetrically in triplicates with a QuAAtro autoanalyser (Seal Analytical, Norderstedt, Germany; Stoll *et al*., 2001). The analyser was calibrated with NaHCO_3_ solutions (with a salinity of 35, achieved by addition of NaCl) to achieve concentrations ranging from 1800 to 2300 μmol DIC kg^−1^. CRMs were used for corrections of errors in instrument performance such as baseline drifts (reproducibility ± 8 μmol kg^−1^). Seawater pH_total_ (*n* = 14) was measured potentiometrically with a two‐point calibrated glass reference electrode (IOline; Schott Instruments). An internal TRIS‐based reference standard (Dickson *et al*., 2007) was used to correct for variability in electrode performance (reproducibility ± 0.015 pH units). Following suggestions by Hoppe *et al*. (2012), seawater carbonate chemistry (including pCO_2_) was calculated from TA and pH using CO_2SYS_ (Pierrot *et al*., 2006). The dissociation constants of carbonic acid of Mehrbach *et al*. (1973; refitted by Dickson & Millero, 1987) were used for the calculations. Dissociation constants for KHSO_4_ were taken from Dickson (1990).

### Growth, elemental composition and production rates

Samples for cell counts were fixed with Lugols solution (1% final concentration) and counted on a light microscope (Axio Observer.D1; Zeiss) after 24 h sedimentation time in 10 ml Utermöhl chambers (Hydro‐Bios, Kiel, Germany, > 1700 cells counted per sample). Samples for determination of Chl*a* were filtered onto 0.6 μm glass‐fibre filters (GF/F; Whatman), immediately placed into liquid nitrogen and stored at −80°C until analysis. Chl was subsequently extracted in 8 ml 90% acetone (2–3 h at 4°C). After removal of the filter, concentrations were determined on a fluorometer (TD‐700; Turner Designs, Sunnyvale, CA, USA), using an acidification step (1 M HCl) to determine phaeopigments (Knap *et al*., 1996). Growth rate determinations started 1–2 d after redilution from daily Chl sampling (*n *=* *3) over 4 d (consecutive) within the first 15 min of the dark phase and were calculated as

(Eqn 1) $$\mu =({\text{log}}_{e}{\left[\text{Chl}\right]}_{t2}-{\text{log}}_{e}{\left[\text{Chl}\right]}_{t1})/\Delta t$$

where [Chl]_*t*1_ and [Chl]_*t*2_ denote the Chl concentrations at the sampling days *t*_1_ and *t*_2_, respectively, and Δ*t* is the corresponding incubation time in d.

Particulate organic carbon (POC) and nitrogen (PON) were measured after filtration onto precombusted (15 h, 500°C) glass‐fibre filters (GF/F 0.6 μm nominal pore size; Whatman). Filters were stored at **−**20°C and dried for at least 12 h at 60°C before sample preparation. Analysis was performed using a CHNS‐O elemental analyser (Euro EA 3000; HEKAtech). Contents of POC and PON were corrected for blank measurements and normalized to filtered volume and cell densities to yield cellular quotas. Biogenic silica (BSi) was determined spectrophotometrically after treatment with a molybdate solution as described in Koroleff (1983). Production rates of Chl, POC, PON and BSi were calculated by multiplying the cellular quota with the growth rate of the respective culture. In order to diminish possible short‐term effects arising from changes in irradiance fields in the dynamic treatments, all samples were taken within the first 30 min of the dark phase.

### Chl‐specific net primary production

Chl‐specific net primary production (NPP) rates were determined in triplicate by incubation of 20 ml of culture with 20 μCi NaH^14^CO_3_ spike (53.1 mCi mmol^−1^; Perkin Elmer, Waltham, MA, USA) in 20 ml glass scintillation vials for 24 h under experimental conditions. From these incubations, 0.1 ml aliquots were immediately removed, mixed with 15 ml of scintillation cocktail (Ultima Gold AB; PerkinElmer) and counted after 2 h with a liquid scintillation counter (Tri‐Carb 2900TR; PerkinElmer) to determine the total amount of added NaH^14^CO_3_ (DPM_100%_). For blank determination (DPM_0%_), one replicate was immediately acidified with 0.5 ml of 6 M HCl. After 24 h of incubation, ^14^C incorporation was stopped by adding 0.5 ml of 6 M HCl to each vial. The entire sample was then left to degas and dry in a custom‐built chamber. When samples were completely dry (1–2 d), 5 ml milli‐Q water were added to resuspend the sample. Subsequently, 15 ml of scintillation cocktail (Ultima Gold AB; PerkinElmer) were added and samples were measured after 2 h with a liquid scintillation counter (Tri‐Carb 2900TR; PerkinElmer). NPP rates (μg C (μg Chl)^−1^ d^−1^) were calculated as

(Eqn 2) $$\text{NPP}=\left(\left[\text{DIC}\right]\times \left({\text{DPM}}_{\mathrm{sample}}-{\text{DPM}}_{0\%}\right)\times 1.05\right)/\left({\text{DPM}}_{100\%}\times t\times \left[\text{Chl}\right]\right)$$

where [DIC] and [Chl] denote the concentrations of DIC and Chl in the sample, respectively. DPM_sample_ denotes the disintegrations min^–1^ (DPM) in the samples, DPM_0%_ reflects the blank value, DPM_100%_ denotes the DPM of the total amount of NaH^14^CO_3_ added to the samples, and *t* is the duration of the incubation.

### Variable Chl fluorescence

Photophysiological characteristics, based on photosystem II (PSII) variable Chl fluorescence, were measured using a fast repetition rate fluorometer (FRRf, FastOcean PTX; Chelsea Technologies, West Molesey, UK) in combination with a FastAct Laboratory system (Chelsea Technologies). The excitation wavelength of the fluorometer's light‐emitting diodes (LEDs) was 450 nm, and the applied light intensity was 1.3 × 10^22^ photons m^−2^ s^−1^. The FRRf was used in single turnover mode, with a saturation phase comprising 100 flashlets on a 2 μs pitch and a relaxation phase comprising 40 flashlets on a 50 μs pitch. All measurements (*n* = 3) were conducted in a temperature‐controlled chamber at 3 ± 0.3°C.

The minimum (*F*_0_) and maximum Chl fluorescences (*F*_m_) were estimated from iterative algorithms for induction (Kolber *et al*., 1998) and relaxation phase (Oxborough, 2012) after subtraction of a blank value (average of *n *=* *8 measurements) in the middle of the dark phase (i.e. 4 h after offset of light). Maximum quantum yields of PSII (apparent PSII photochemical quantum efficiency; *F*_v_/*F*_m_) were calculated as

(Eqn 3) $$F_{v}/F_{m}=\left(F_{m}-F_{0}\right)/F_{m}$$

Photosystem II electron flux was calculated on a volume basis (JV_PSII_; (mol e^−^ m^−3^ d^−1^)) using the absorption algorithm (Oxborough *et al*., 2012). The JV_PSII_ rates were converted to Chl‐specific absolute rates (ETR (mol e^−^ (mol Chl)^−1^s^−1^)) by dividing it by the Chl concentration of the sample at the time point of the measurement and the number of seconds per day. Chl‐specific JV_PSII_‐based photosynthesis–irradiance (PI) curves were conducted four times a day (1 and 8 h after the onset of light as well as directly after and 4 h after the onset of darkness) at 15 irradiance (I) intensities between 6 and 650 μmol photons m^−2^ s^−1^, with an acclimation time of 90 s per light step. Following the suggestion by Silsbe & Kromkamp (2012), the light‐use efficiency (*α*), and the light saturation index (*I*_K_) were estimated by fitting the data to the model by Webb *et al*. (1974):

(Eqn 4) $$\text{ETR}=\alpha \times I_{K}\times \left[1-e^{\left(-I\times I_{K}\right)}\right]$$

The maximum electron transport rates (ETR_max_ (mol e^−^ mol^–1^ Chl s^−1^) were estimated after applying a beta phase fit as described by Oxborough (2012). Daily electron transport rates (ETR_24 h_ (mol e^−^ (mol Chl)^−1^d^−1^)) were estimated by integrating the number of electrons transported over the 16 h light phase. ETR_24 h_ were calculated in 5 min steps of *I*‐values of both light regimes (i.e. 90 μmol photons m^−2^ s^−1^ under constant and variable irradiances under dynamic light) using *α*,* I*_K_ and ETR_max_ from the PI curve measured closest to the time point of interest. Chl concentrations for normalizations were corrected using the growth rate and the time difference between FRRf and Chl measurements. To estimate the energy transfer efficiency from photochemistry to biomass build‐up, the electron requirement for carbon fixation (Φ_e,C_ (mol e^−^ (mol C)^–1^)) was calculated for each treatment by dividing the ETR_24 h_ by NPP (expressed as molar quantities). It should be noted that differences in the spectral composition of the light used for ETR (i.e. blue light) and NPP measurements (i.e. white light) could lead to a systematic overestimation of Φ_e,C_.

Nonphotochemical quenching of Chl fluorescence (NPQ) at irradiances of 490 and 650 μmol photons m^−2^ s^−1^ (i.e. the maximum irradiance applied in the dynamic light cycle as well as the maximum irradiance step of the PI curve) were calculated using the normalized Stern–Volmer coefficient (also termed NSV) as described in Oxborough (2012) and McKew *et al*. (2013):

(Eqn 5) $$\left(F_{q}^{\prime }/F_{v}^{\prime }\right)-1=F_{0}^{\prime }/F_{v}^{\prime }$$

where $F_{0}^{\prime }$ was measured after each light step (with a duration of 90 s).

### Statistics

All data are given as the means of the replicates ± 1 SD. To test for significant differences between the treatments, two‐way ANOVAs with additional normality (Shapiro–Wilk) and *post hoc* (Holm–Sidak method) tests were performed. The significance level was set to 0.05. Statistical analyses were performed using the program SigmaPlot (SysStat Software Inc., San Jose, CA, USA).

## Results

### Growth rates and elemental composition

**Figure 2:**
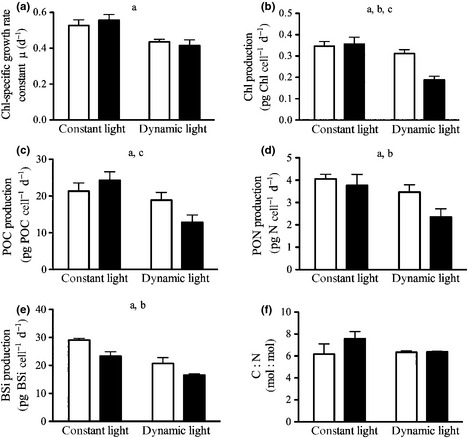
Chlorophyll‐specific growth rate constants (a), production rates of Chl (b), particulate organic carbon (POC) (c), particulate organic nitrogen (PON) (d), BSi (e) and cellular C : N ratios (f) of *Chaetoceros debilis* at pCO_2_ concentrations of 390 μatm (open bars) and 1000 μatm (closed bars) under constant and dynamic light regimes (*n *=* *3; mean ± 1 SD). Letters indicate significant (*P *<* *0.05) differences between: a, light treatments; b, pCO_2_ treatments; c, significant interactions between light and pCO_2_ treatments.

Chl‐specific growth rates (Fig. 2a; Table 2) under constant light conditions were similarly high, being 0.53 ± 0.03 and 0.56 ± 0.03 d^−1^ in ambient and high pCO_2_ treatments, respectively. Under dynamic light, growth rates were significantly lower than under constant light (ANOVA, *F *=* *51; *P *<* *0.001; Table S1). Also under these conditions, growth rates were unaffected by the applied pCO_2_ treatments, being 0.44 ± 0.01 d^−1^ at ambient pCO_2_ and 0.42 ± 0.03 d^−1^ at high pCO_2_.

**Table 2:**
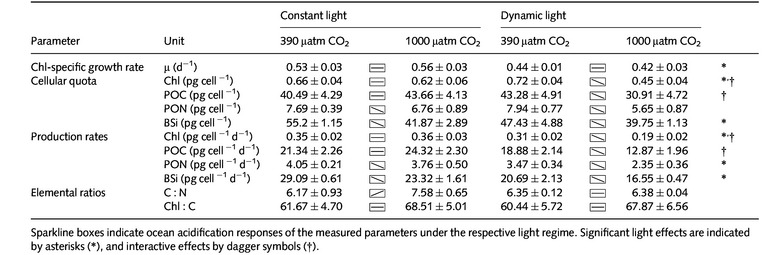
Chlorophyll‐specific growth rates, cellular quotas and production rates of Chl, particulate organic carbon (POC), particulate organic nitrogen (PON) and biogenic silica (BSi) of *Chaetoceros debilis* (*n *=* *3; mean ± 1 SD) under two pCO_2_ concentrations at constant and dynamic light regimes

With respect to the amount of Chl per cell (Table 2), we observed significant effects of both pCO_2_ (ANOVA, *F *=* *28; *P *<* *0.001) and light treatments (ANOVA, *F *=* *6; *P *=* *0.047). Under dynamic light, Chl quotas significantly decreased with increasing pCO_2_ (*post hoc*,* P *<* *0.001), while they remained unaffected by OA under constant light, leading to a significant interactive effect of pCO_2_ and light intensity on cellular Chl quotas (ANOVA, *F *=* *21; *P *=* *0.002). Similarly, the production of Chl per cell (Fig. 2b; Table 2) was also significantly affected by both pCO_2_ (ANOVA, *F *=* *18; *P *=* *0.003) and light (ANOVA, *F *=* *56; *P *<* *0.001). Both factors also had an interactive effect on production rates (ANOVA, *F *=* *25; *P *=* *0.001), which led to a significant decrease in Chl production under dynamic light and increasing pCO_2_ (*post hoc*,* P *<* *0.001). The ratio of Chl : C (Table 2) was not significantly affected by any treatment.

Cellular quotas of POC (Table 2) under constant light did not differ between ambient and high pCO_2_, whereas they significantly decreased with increasing pCO_2_ under dynamic light (*post hoc* test, *P *=* *0.010; significant ANOVA interaction between pCO_2_ and light, *F *=* *9; *P *=* *0.018). Overall, POC production (Fig. 2c, Table 2) under constant light was not significantly affected by pCO_2_, but was significantly reduced under dynamic vs constant light (ANOVA, *F *=* *31; *P *<* *0.001). Under dynamic light conditions, POC production also significantly decreased with increasing pCO_2_ (*post hoc*,* P *=* *0.009), resulting in a significant interaction term between pCO_2_ and light conditions (ANOVA, *F *=* *9; *P *=* *0.018).

Cellular quotas of PON (Table 2) were significantly reduced under high vs ambient pCO_2_ (ANOVA, *F *=* *14; *P *=* *0.006), irrespective of the light conditions applied. Also the production of PON (Fig. 2d; Table 2) decreased significantly with decreasing pCO_2_ (ANOVA, *F *=* *11; *P *=* *0.010). PON production was significantly higher under constant than under dynamic light (ANOVA, *F *=* *23; *P *=* *0.001). Under constant light, C : N ratios significantly increased with increasing pCO_2_ (Fig. 2f; Table 2; *post hoc*,* P *=* *0.017). Under dynamic light, no such response was observed. Significant differences in C : N ratios between the light treatments were observed under high pCO_2_ only, where dynamic light led to an decrease in C : N (*post hoc*,* P *=* *0.033).

Cultures exhibited a highly significant decline in the cellular quota of biogenic silica (BSi; Table 2) with increasing pCO_2_ (ANOVA, *F *=* *38; *P *<* *0.001). BSi quotas were furthermore lower under dynamic than under constant light (ANOVA, *F *=* *9; *P *=* *0.020). We also observed a highly significant decrease in BSi production (Fig. 2e; Table 2) with increasing pCO_2_ (ANOVA, *F *=* *38; *P *<* *0.001). Furthermore, BSi production was significantly lower under dynamic than under constant light (ANOVA, *F *=* *90; *P *<* *0.001).

### Chl‐specific NPP

**Figure 3:**
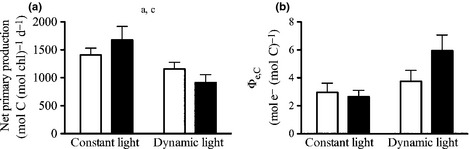
Chlorophyll‐specific net primary production (NPP; a) and electron requirement for carbon fixation (Φ_e,C_; b) at pCO_2_ concentrations of 390 μatm (open bars) and 1000 μatm (closed bars) under constant and dynamic light regimes (*n *=* *3; mean ± 1 SD). Letters indicate significant (*P *<* *0.05) differences between: a, light treatments; b, pCO_2_ treatments; c, significant interactions between light and pCO_2_ treatments.

Chl‐specific NPP (Fig. 3; Table 3) under constant light increased slightly, yet insignificantly, with increasing pCO_2_. Under dynamic light, NPP was lower than under constant light (ANOVA, *F *=* *27; *P *<* * 0.001; Table S2). Under these conditions, NPP was also significantly decreased with increasing pCO_2_ (*post hoc*,* P *<* *0.001), resulting in a significant interaction between pCO_2_ and light conditions (ANOVA, *F *=* *7; *P *=* *0.028).

**Table 3:**
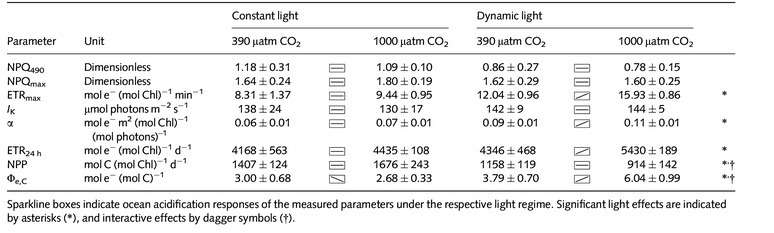
Fast repetition rate (FRR)‐flourometrical photosystem II (PSII) photochemistry measurements – nonphotochemical quenching at 490 μmol photons m^−2^ s^−1^ (NPQ_490_), maximal NPQ (NPQ_max_), maximal absolute electron transfer rates through PSII (ETR_max_), light saturation index (*I*_K_) and the maximum light‐use efficiency (initial slope *α*) at night (4 h after the onset of darkness) as well as integrated daily ETR (ETR_24 h_), net primary production (NPP) and energy transfer efficiency from photochemistry to biomass production (Φ_e,C_) under two pCO_2_ concentrations at constant and dynamic light regimes (*n *=* *3; mean ± 1 SD)

### Chl fluorescence‐based photophysiology

The dark‐acclimated quantum yield efficiency of PSII (*F*_v_/*F*_m_) was similar in all treatments, with values of 0.53 ± 0.01. Neither nonphotochemical quenching (NPQ) at 490 μmol photons m^−2^ s^−1^ nor maximal NPQ at 650 μmol photons m^−2^ s^−1^ (NPQ_max_, Table 3) was significantly affected by the applied treatments (Supporting Information, Fig. S1).

**Figure 4:**
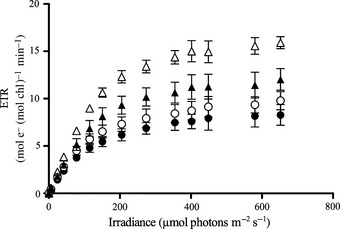
Night‐time responses in Chl‐specific electron transport rate (ETR) to increasing irradiance from constant (closed circles) and dynamic light treatments (closed triangles) at 390 μatm pCO_2_ as well as from constant (open circles) and dynamic light treatments (open triangles) at 1000 μatm pCO_2_ (*n* = 3; mean ± 1 SD).

The fitted parameters of night‐time FRRf‐based PI curves (Fig. 4) were strongly influenced by experimental treatments. The maximal electron transport rates through PSII (ETR_max_; Table 3) increased with increasing pCO_2_ (ANOVA, *F *=* *17; *P *=* *0.003) and were also significantly higher under dynamic than under constant light (ANOVA, *F *=* *71; *P *<* *0.001). *Post hoc* tests revealed that the OA response was much more pronounced under dynamic light (*post hoc*,* P *=* *0.022) than under constant light (*post hoc*,* P *=* *0.222). No sign of photoinhibition of ETR was observed (Fig. 4). The maximum PSII light‐use efficiency (*α*; Table 3) was significantly higher under OA than under ambient pCO_2_ (ANOVA, *F *=* *14, *P *=* *0.006), a result that was mainly driven by the responses under dynamic light (*post hoc*,* P *<* *0.001) and not that pronounced under constant light (*post hoc*,* P *=* *0.120). In addition, *α*‐values in both pCO_2_ treatments were significantly higher under dynamic than under constant light (ANOVA, *F *=* *35; *P *<* *0.001). The PSII light saturation point (*I*_K_; Table 3) was not significantly affected by the experimental treatments.

Similarly, cumulative electron transport rates over 24 h (ETR_24 h_; Table 3) were also higher under OA than under ambient pCO_2_ (ANOVA, *F *=* *9; *P *=* *0.029) as well as under dynamic vs constant light (ANOVA, *F *=* *7; *P *=* *0.015). The strongest responses were observed under high pCO_2_ (*post hoc*,* P *=* *0.013) and dynamic light (*post hoc*,* P *=* *0.008). The electron requirement for carbon fixation (Φ_e,C_; Fig. 3; Table 3) was significantly higher under dynamic than under constant light (ANOVA, *F *=* *28; *P *<* *0.001). In the dynamic light treatments, Φ_e,C_ was also much higher under OA compared with ambient pCO_2_ (*post hoc*,* P *<* *0.001). Such an OA response was not observed under constant light conditions, where Φ_e,C_ decreased slightly (*post hoc*,* P *=* *0.047). Overall, these responses led to a significant effect of pCO_2_ (ANOVA, *F *=* *6; *P *<* *0.035) as well as – because of the opposing OA trends under the two light treatments – a significant interaction term between light treatments and pCO_2_ concentrations (ANOVA, *F *=* *10; *P *<* *0.012).

## Discussion

### Dynamic light exerts high metabolic costs

Prevailing strong winds lead to deeply mixed surface layers and highly dynamic light regimes in the SO (Nelson & Smith, 1991). Phytoplankton species occurring in this environment can therefore be expected to cope well with dynamic light conditions. In fact, cellular POC and PON quotas as well as C : N and Chl : C at 390 μatm pCO_2_ did not differ between the light treatments in *C. debilis* (Table 2). The maintenance of cellular stoichiometry under dynamic light was, however, achieved at the expense of growth and biomass build‐up (Figs 2, 3; Table 2). A decline in growth rates under dynamic light is an overarching pattern observed in several studies (van de Poll *et al*., 2007; Mills *et al*., 2010; Boelen *et al*., 2011; Shatwell *et al*., 2012). Moreover, in the current study, Chl fluorescence‐based estimates of the light‐use efficiency *α* and ETR_max_, as well as daily integrated ETR_24 h_, were significantly higher under dynamic than under constant light (Table 3), while NPP and biomass build‐up under dynamic light were significantly lowered (Figs 2, 3). This implies that under dynamic light, the overall energy transfer efficiency from photochemistry to net biomass production was substantially reduced (Wagner *et al*., 2006; Ihnken *et al*., 2011; Su *et al*., 2012; Jin *et al*., 2013).

The electron requirement of carbon fixation (Φ_e,C_) was indeed significantly higher under dynamic light conditions (Fig. 3), hinting at an increase in other electron‐consuming processes such as mitochondrial respiration, photorespiration or alternative electron cycling (Prásil *et al*., 1996; Badger *et al*., 2000; Wagner *et al*., 2006; Waring *et al*., 2010; Thamatrakoln *et al*., 2013). While Φ_e,C_ should theoretically be 4–6 mol e^−^ (mol C)^−1^ (Genty *et al*., 1989; Suggett *et al*., 2009), the estimates for Φ_e,C_ in this study range between *c*. 3 and 6 mol e^−^ (mol C)^−1^ (Table 3). Values between 1.2 and 54.3 mol e^−^ (mol C)^−1^ have been previously observed in field studies and laboratory experiments (Suggett *et al*., 2009; Lawrenz *et al*., 2013). Values of Φ_e,C_ < 4 mol e^−^ (mol C)^−1^ have been attributed to systematic errors in the ETR calculations (Lawrenz *et al*., 2013). In addition, differences in temporal scales between measures (e.g. Kromkamp & Forster, 2003) as well as short acclimation times may lead to a systematic underestimation of ETRs. Regarding the latter, directional errors in Φ_e,C_ are, however, unlikely to have contributed to the differences between light regimes, as the treatment‐specific variations in α, *I*_K_ and ETR_max_ were similar at different time points (data not shown). Irrespective of a potential underestimation of Φ_e,C_, the observed trends indicate comparably low energy transfer efficiency under dynamic light, which could well explain the observed decrease in growth and NPP compared with constant light (Fig. 3). This interpretation is further corroborated by the observed *I*_K_‐independent behaviour of the PI curves (i.e. changes in ETR_max_ and *α* while *I*_K_ stays constant; Table 3), which can be attributed to changes in processes that decouple carbon fixation from photosynthetic electron transport through the consumption of ATP and reductants (Behrenfeld *et al*., 2004, 2008).

Diatom cells growing under dynamic light need to adjust their photosynthetic apparatus to achieve a balance between photoprotection at high light and effective light‐harvesting at low light. No high‐light stress was observed in ETR vs irradiance curves (Fig. 4), indicating successful photoprotection under all tested scenarios. In line with other studies on dynamic light (van de Poll *et al*., 2007; Kropuenske *et al*., 2009; Alderkamp *et al*., 2012; Su *et al*., 2012), we also did not observe an increase in NPQ capacity (Table 3). Successful photoprotection may be achieved via other processes such as increased connectivity between reaction centres (Trimborn *et al*., 2014) or the induction of alternative electron pathways (e.g. Mehler reaction, electron flow around PSII or PSI) that can supplement the xanthophyll cycle in diatoms (Prásil *et al*., 1996; Asada, 1999; Waring *et al*., 2010). These mechanisms could have contributed to the observed increase in Φ_e,C_ (Fig. 3) under dynamic vs constant light.

In addition, the apparent insensitivity of electron transport towards high‐light stress (Fig. 4) does not mean that no photodamage of reaction centres occurs. In fact, an uncoupling between PSII inactivation and the rate of electron flow has been described as a common mechanism for phytoplankton under natural light regimes (Behrenfeld *et al*., 1998). The uncoupling can be explained by the presence of ‘excess PSII capacity’ (i.e. more reaction centres than are actually needed), allowing for high photochemical efficiencies even if light‐dependent photoinactivation of PSII increases (Behrenfeld *et al*., 1998). This overproduction and subsequent repair of PSII, including the susceptible D1 subunit and associated proteins, imposes high metabolic costs for the phytoplankton cell (Raven, 2011). Whether or not the costs, being associated with the high‐light phases of the dynamic light treatment, are compensated for by the subsequent period of low light depends on the rates of both, the changes in light intensity and D1 repair (Behrenfeld *et al*., 1998; Marshall *et al*., 2000). In the tested scenarios here, we did not observe the manifestation of photoinhibition (Fig. 4). Therefore, we postulate that a large fraction of the decline in growth and energy transfer efficiency from photochemistry to biomass production under dynamic light (Fig. 3; Table 3) results from increased metabolic costs of photoprotection and elevated D1 turnover at high light in combination with the consequences of light limitation in the low‐light phases.

### Ocean acidification increases energy‐use efficiency under constant light

Changes in CO_2_ supply have been shown to differentially affect SO diatoms on the species level (Boelen *et al*., 2011; Hoogstraten *et al*., 2012a,b; Trimborn *et al*., 2013, 2014) as well as in natural communities (Tortell *et al*., 2008; Feng *et al*., 2010; Hoppe *et al*., 2013). The Antarctic diatom *C. debilis* was shown to exhibit increased energy‐use efficiencies (i.e. higher growth rates, but lower O_2_ evolution) as well as decreased dark respiration under high pCO_2_ and constant light (Trimborn *et al*., 2013, 2014). In the present study, C : N ratios were higher under OA and constant light, while growth rates and NPP of *C. debilis* were only slightly stimulated under these conditions (Figs 2, 3; Tables 2, 3). The differences in the CO_2_ sensitivity of *C. debilis* most likely originate from different pCO_2_ treatments applied in the two studies, as significant changes were mainly observed between intermediate (ambient) and low (glacial) pCO_2_ concentrations, the latter not being investigated in the current study. Similar to our results, two other species of *Chaetoceros* also showed little or no growth response to OA, but these results apparently also depended on the applied light intensities (Boelen *et al*., 2011; Ihnken *et al*., 2011). In CO_2_ manipulation experiments with SO phytoplankton communities, however, *Chaetoceros* was found to benefit from elevated pCO_2_, as this genus dominated the applied OA treatments (Tortell *et al*., 2008; Feng *et al*., 2010).

Such OA responses have often been attributed to the mode of CCMs, which can differ in the ability to reach rate saturation and to respond to environmental changes as well as in the associated costs of these processes. In the case of diatoms, CCMs have been shown to be very effective avoiding carbon limitation, but also to be regulated as a function of external CO_2_ concentration (e.g. Raven & Johnston, 1991; Trimborn *et al*., 2009; Hopkinson *et al*., 2011). Elevated pCO_2_ often leads to a down‐regulation of CCM activity, thereby reducing the overall costs of carbon acquisition (Burkhardt *et al*., 2001; Rost *et al*., 2003; Hopkinson *et al*., 2011). Even though Trimborn *et al*. (2013) observed a rather constitutively expressed CCM for *C. debilis*, it can be speculated that the higher gross CO_2_ uptake under elevated pCO_2_ may have contributed to the observed stimulation in growth. In conclusion, the often documented beneficial OA effects at constant light could, to a large degree, be explained by overall lowered costs of the CCM.

Photoacclimation can also be influenced by CO_2_ via the CO_2_‐dependent regulation of CCMs and RubisCO concentrations, as these properties can affect the amount of electrons being used during carbon fixation (Tortell, 2000; Rost *et al*., 2006; Reinfelder, 2011). Trimborn *et al*. (2014) showed strong effects of short‐term exposure to low pCO_2_ concentrations on various photophysiological parameters in *C. debilis*. In line with findings on two *Thalassiosira* species (McCarthy *et al*., 2012), ETR_max_ increased with increasing pCO_2_ (Table 3), indicating higher substrate saturation at RubisCO and higher activities of the Calvin cycle under OA and constant light. With respect to the balance between light and dark reaction of photosynthesis, we observed a slight decrease in Φ_e,C_ with increasing pCO_2_ (Fig. 3; Table 3). These results could imply that the Calvin cycle acts as a better energy sink under elevated pCO_2_ and constant light, as has been proposed by Trimborn *et al*. (2014). Such an increase in electron‐use efficiency could thus explain the beneficial effects of OA (Figs 2, 3).

### Dynamic light reverses the responses to ocean acidification

In line with previous findings on *Chaetoceros* (Boelen *et al*., 2011; Ihnken *et al*., 2011), we observed slight, yet insignificant, enhancement in growth, POC production and NPP with increasing pCO_2_ under constant light (Figs 2, 3). In other studies, growth and NPP of *Chaetoceros* were strongly stimulated under elevated pCO_2_ (Tortell *et al*., 2008; Feng *et al*., 2010; Hoppe *et al*., 2013; Trimborn *et al*., 2013). When comparing these trends with the OA responses from the dynamic light treatments, a completely different picture emerges: POC production and NPP decrease under OA by *c*. 30 and 50%, respectively. The putatively beneficial effects of elevated pCO_2_ seem not only to be dampened, but even reversed under dynamic light as cells significantly slow down biomass production (Fig. 2). Boelen *et al*. (2011) did not observe any significant responses of *C. brevis* to either OA or dynamic light. Their results indicate a low sensitivity of this strain to increasing pCO_2_ up to 750 μatm, even though POC production under OA was reduced by *c*. 15% in their dynamic high‐light treatment. In line with our study, Jin *et al*. (2013) observed a decline in carbon fixation rates of the coccolithophore *G. oceanica* under OA and short‐term exposure to dynamic light.

Surprisingly, the decline in biomass build‐up in this study was observed even though electron transport through PSII was most efficient under these conditions (Table 3). In addition, there was no sign of photoinhibition after short‐term exposure to irradiances up to 650 μmol photons m^−2^ s^−1^ in any of the treatments (Fig. 4). At higher irradiances, however, rETRs in *C. debilis* were found to decrease (Trimborn *et al*., 2014). The photophysiological results therefore suggest that the excess capacity of photosynthesis (Behrenfeld *et al*., 1998) was sufficient to prevent chronic photoinhibition under the applied assay irradiances (Fig. 4). As these photophysiological results do not explain the decline in POC production and NPP observed under OA and dynamic light (Figs 2, 3) and as the observed *I*_K_‐independent changes in the PI curves (Table 3) are indicative of changes in the demand and distribution of energy and reductive equivalents (Behrenfeld *et al*., 2004, 2008), the underlying reason may be associated with an imbalance between light and dark reactions of photosynthesis.

There is increasing evidence that diatoms are more susceptible to D1 inactivation and photoinhibition under OA than under ambient pCO_2_ concentrations (Wu *et al*., 2010; Gao *et al*., 2012a; McCarthy *et al*., 2012). Li & Campbell (2013) observed that under OA, *Thalassiosira pseudonana* has enhanced growth rates under low, but not high light, a finding that is in line with studies on cyanobacteria and coccolithophores (Kranz *et al*., 2010; Rokitta & Rost, 2012). As photosynthesis shifts progressively from light towards carbon limitation under increasing irradiance, CCM activity also needs to be increased under these conditions (Beardall, 1991; Rost *et al*., 2006). The CCM, however, is typically down‐regulated under OA (Burkhardt *et al*., 2001; Rost *et al*., 2003), which could restrict the capacity to rapidly sink more electrons in the Calvin cycle or to drain excess energy by HCO_3_^−^ cycling under short‐term high‐light stress (Tchernov *et al*., 1997; Rost *et al*., 2006). This could result in a lower capability to cope with high light and could increase photoinactivation of PSII under OA (Beardall & Giordano, 2002; Ihnken *et al*., 2011; Gao *et al*., 2012b), shifting the susceptibility to photoinhibition towards lower irradiances. The proposed mechanism implies that, under dynamic light, cells exposed to higher pCO_2_ concentrations experience high‐light stress for longer time periods compared with cells grown under ambient pCO_2_. Under constant light, no photoacclimation to high‐light phases would be needed, so that an OA‐induced surplus of energy could be directly used to build more biomass (Figs 2, 3; Tortell *et al*., 2008; Trimborn *et al*., 2013). Under dynamic light, however, this extra energy may lead to higher metabolic costs for photoacclimation and D1 repair during high‐light phases, which apparently cannot be compensated by lowered operational costs of CCMs. The mechanism proposed here could collectively explain the observed higher demand for energy and reductive equivalents (i.e. *I*_K_‐independent increase in ETR_max_, Table 3; Behrenfeld *et al*., 2004), as well as the decline in NPP under OA and dynamic light, ultimately leading to a strong increase in Φ_e,C_ (Fig. 3).

Under the conditions applied here, *C. debilis* seems to be able to circumvent measurable photoinhibition, even though we speculate that this comes at a high cost, especially under OA combined with dynamic light. Under higher pCO_2_ concentrations as well as higher average or more dynamic irradiances, however, OA could induce measurable damage to the photosynthetic apparatus in addition to presumably high metabolic costs incurred from D1 turnover and photosystem repair (Raven, 2011; Li & Campbell, 2013). Therefore, the modulation of OA responses probably also varies depending on the light regime applied (cf. Boelen *et al*., 2011). Further, the response pattern may be modulated by depth‐dependent changes in the spectral composition of light (Falkowski & LaRoche, 1991), which were not investigated in the present study. In view of the generally high plasticity of photoacclimation in diatoms (Wagner *et al*., 2006; Lavaud *et al*., 2007), the interactive effects described here might be even more pronounced in other phytoplankton taxa. In any case, our data have demonstrated that a combination of OA and dynamic light could impose significantly more stress onto phytoplankton than was previously thought.

### Implications for ecology and biogeochemistry

In summary, dynamic light was shown to drastically alter OA effects on an ecologically important SO diatom species, leading to a strong decline in primary production under OA and dynamic light. The observed response patterns can be explained by changes in the cellular energetic balance, as the energy transfer efficiency from photochemistry to biomass production was drastically reduced under OA and dynamic light. Given the increasing number of studies dealing with possible interactive effects of OA with high‐light stress on phytoplankton (e.g. Wu *et al*., 2010; Gao *et al*., 2012a; Gao & Campbell, 2014), the importance of excess PSII capacity should be investigated in future.

Our results also have important implications for the current understanding of OA effects on marine phytoplankton. As has been shown for several environmental variables, such as temperature (e.g. Tatters *et al*., 2013) or nutrient concentrations (e.g. Fu *et al*., 2010; Hoppe *et al*., 2013), interactive effects need to be considered when predicting future productivity and ecosystem functioning. As a central feature of oceanic environments, dynamic light is an especially important aspect (Mitchell *et al*., 1991; MacIntyre *et al*., 2000), which has been neglected in most OA studies so far. If our results are representative, the often proposed ‘CO_2_ fertilization’ may be dampened or even reversed in many natural environments. In this context, it is important to consider that anthropogenic CO_2_ emissions do not only lead to OA, but also to a warming of the surface ocean (Sarmiento *et al*., 2004). A concomitant shoaling of the upper mixed layer would thus change the integrated intensity and variability of the light regimes encountered by phytoplankton cells (Rost *et al*., 2008; Steinacher *et al*., 2010), making the interactive relationship between OA and light regimes even more important to consider.

Regarding the SO, it seems likely that synergistic effects of iron limitation and dynamic light, both dominant features of this region (Boyd, 2002; de Baar *et al*., 2005; Alderkamp *et al*., 2012), jointly lower the potential benefits of OA. Under iron‐enriched conditions, as in the present study, diatom taxa such as *Chaetoceros* and *Fragilariopsis* have been shown to dominate OA treatments under constant light, suggesting a higher potential for export production (Tortell *et al*., 2008; Hoppe *et al*., 2013). The lowered NPP under OA and dynamic light, however, questions the reliability of such predictions. The aspect of ballasting also has to be considered, as siliceous frustules make diatoms efficient vectors for carbon (Sarthou *et al*., 2005). In line with Milligan *et al*. (2004), we observed a decline in both cellular BSi quotas and production rates with increasing pCO_2_ (Fig. 2; Table 2), which further argues against a stimulation of the biological carbon pump. To date, the effects of dynamic light on OA responses and the underlying reasons for them, as observed in this study, were unknown. This new knowledge will change our perception of phytoplankton under climate change.

## Supporting information

Please note: Wiley Blackwell are not responsible for the content or functionality of any supporting information supplied by the authors. Any queries (other than missing material) should be directed to the *New Phytologist* Central Office.

**Fig. S1** Night‐time development of nonphotochemical quenching (NPQ) with increasing irradiance under the different treatment conditions.
**Table S1** Results from two‐way ANOVAs for all measured acclimation parameters
**Table S2** Results from two‐way ANOVAs for Chl fluorescence‐based parameters, net primary production and the electron requirement for carbon fixation
