# Supplementary material for: Ocean acidification decreases the light‐use efficiency in an Antarctic diatom under dynamic but not constant light
Source: New Phytol. 2015 Feb 24;207(1):159–71. doi: 10.1111/nph.13334 (PMC4950296; doi:10.1111/nph.13334)
Supplement: Supplementary file 1 — Fig. S1 Night‐time development of nonphotochemical quenching (NPQ) with increasing irradiance under the different treatment conditions. Table S1 Results from two‐way ANOVAs for all measured acclimation parameters Table S2 Results from two‐way ANOVAs for Chl fluorescence‐based parameters, net primary production and the electron requirement for carbon fixation [file NPH-207-159-s001.pdf]

Supporting Information for Hoppe *et al.* 2015):

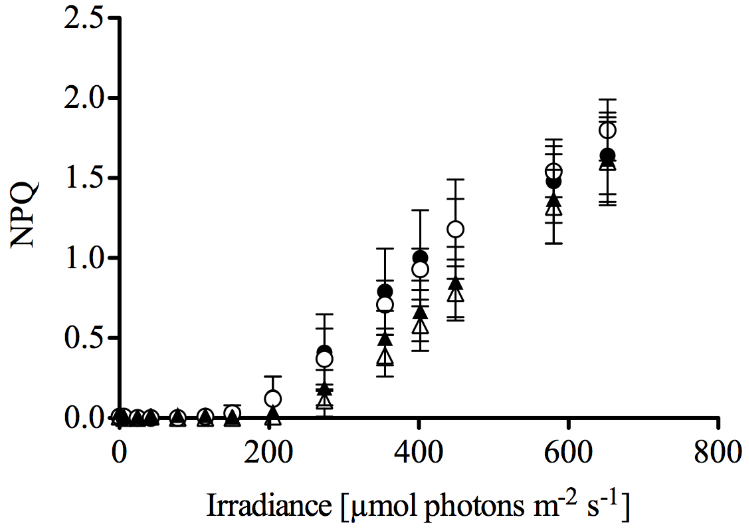

Figure S1: Night time development of non-photochemical quenching (NPQ) with increasing irradiance from constant (filled circles) and dynamic light treatments (filled triangles) at 390 µatm pCO_2 as well as from constant (open circles) and dynamic light treatments (open triangles) at 1000 µatm pCO_2 (n=3).

Table S1: Results from two-way ANOVAs for all measured acclimation parameters. The significance level was set to p values <0.05, significant p values are given in bold.

	**µ**		**POC:PON**		**Chl:POC**	
**Source of Variation**	F	p	F	p	F	p
**light**	50.9	**<0.001**	2.4	0.160	0.2	0.797
**pCO_2**	0.1	0.757	4.8	0.060	2.4	0.158
**light x pCO_2**	2.3	0.164	4.4	0.071	<0.1	0.993

	**Chl $cell^{-1}$**		**POC $cell^{-1}$**		**PON $cell^{-1}$**		**BSi $cell^{-1}$**	
**Source of Variation**	F	p	F	p	F	p	F	p
**light**	5.5	**0.047**	3.7	0.093	1.0	0.358	8.5	**0.020**
**pCO_2**	27.6	**<0.001**	3.1	0.116	13.7	**0.006**	38.1	**<0.001**
**light x pCO_2**	21.0	**0.002**	8.9	**0.018**	2.4	0.158	2.8	0.136

	**Chl $cell^{-1}$ day^{-1}**		**POC $cell^{-1}$ day^{-1}**		**PON $cell^{-1}$ day^{-1}**		**BSi $cell^{-1}$ day^{-1}**	
**Source of Variation**	F	p	F	p	F	p	F	p
**light**	56.2	**<0.001**	30.9	**<0.001**	22.6	**0.001**	89.7	**<0.001**
**pCO_2**	18.0	**0.003**	1.5	0.261	11.1	**0.010**	38.3	**<0.001**
**light x pCO_2**	24.8	**0.001**	12.9	**0.007**	3.8	0.086	1.0	0.339

Table S2: Results from two-way ANOVAs for Chl fluorescence-based parameters, net primary production and the electron requirement for carbon fixation. The significance level was set to p values <0.05, significant p values are given in bold.

	ETRmax		alpha		I_K	
**Source of Variation**	F	p	F	p	F	p
**light**	70.5	**<0.001**	35.4	**<0.001**	1.0	0.344
**pCO_2**	17.1	**0.003**	13.6	**0.006**	0.2	0.700
**light x pCO_2**	5.1	0.054	1.5	0.256	0.3	0.608

**Source of Variation**	ETR_{24h}		NPP		Φe,C	
	F	p	F	p	F	p
**light**	7.1	**0.029**	26.6	**<0.001**	28.3	**<0.001**
**pCO_2**	9.4	**0.015**	<0.1	0.905	6.4	**0.035**
**light x pCO_2**	3.4	0.101	6.9	**0.028**	10.4	**0.012**

	NPQ_{490}		NPQ_{max}	
**Source of Variation**	F	p	F	p
**light**	0.2	0.694	<0.1	0.852
**pCO2**	4.9	0.058	0.4	0.564
**light x pCO2**	0.1	0.821	0.1	0.779
